# Histological changes in intra-oral skin flaps

**DOI:** 10.1186/1758-3284-1-2

**Published:** 2009-01-12

**Authors:** Julia Anne Woolgar, Asterios Triantafyllou

**Affiliations:** 1 Oral Pathology, School of Dental Sciences and Dental Hospital, University of Liverpool, Pembroke Place, Liverpool L3 5PS, UK

## Abstract

This review outlines the histological features of intra-oral skin flaps and their pathology as encountered in a routine head and neck diagnostic histopathology service. Problems in recognising and interpreting unfamiliar and complex appearances, and areas of diagnostic and prognostic uncertainty, are highlighted.

## Introduction

Free tissue transfer using vascularised skin flaps such as the radial forearm flap has become a routine part of the management and rehabilitation of patients with squamous cell carcinoma of the mouth and oropharynx (OSCC) [1,2]. How the skin flap adapts to the oral environment has been researched in animal models [3] and in humans [4-10] with recent articles focusing on general performance status, sensory function, particular histological features and immunohistochemically assessed changes in the cornification process [11-13]. Grossly, the flaps often appear similar to the native oral mucosa [7] and a gradual "mucosalisation" of the transferred skin with loss of the stratum corneum and shrinkage of pilosebaceous units has been suggested [3,8]. The notion of "mucosalisation" has, however, been challenged [4] and recent articles suggest that the cutaneous phenotypes are maintained unless there is chronic candidiasis [9,12]; even when the more "alien" jejunal mucosa is transferred within the mouth, preservation of its phenotype is long-term [14]. Such continued distinction between transferred and native tissues is not unexpected as it is the underlying stroma that influences the phenotype of the overlying epithelium [15]. Many clinicians regard candidal infestation of the epidermis as an inevitable consequence of the wet oral environment and a frequent cause of clinical lesions [5,6,8,16]. For example, chronic candidiasis was evident in 75% of punch biopsies in the study on the adaptive properties of the transferred skin reported by Katou et al [6]. Hence, chronic candidiasis is often the preferred clinical diagnosis when localised erythematous, leukoplakic and erosive/ulcerative lesions are noted at the junction of the skin flap and the oral mucosa. Nevertheless, cancerous and precancerous lesions should always be considered in the clinical differential diagnosis since a study on survival and patterns of recurrence in 200 OSCC patients indicated that 5% of the patients developed a new primary tumour at the skin flap/mucosal junction [17]. The clinical diagnostic uncertainty means that the management of such lesions usually involves biopsy and histological assessment. The latter, however, often presents difficulties. Information that may facilitate the pathologist's interpretation of the complex histological features of chronic candidiasis of the transferred skin is not readily available and the biopsy may be submitted with a complicated clinical history (such as widespread or multifocal dysplasia of the remaining oral mucosa, or previous radiotherapy), thus compounding the difficulty.

The present review aims to comment on the range of histopathological changes seen in skin flap biopsies, highlighting diagnostic difficulties and potential pitfalls. It is based on long-term experience gained on abundant routine diagnostic material and concentrates on the morphology and possible pathogenesis of lesions rather than their incidence and frequency. To begin, the histological features of skin flaps removed as part of subsequent reconstructive surgery will be described to illustrate "normal histological features", followed by an account of a range of pathological lesions including chronic candidiasis, squamous epithelial dysplasia and carcinoma, and miscellaneous acanthomas and adnexal tumours.

## "Normal histological features"

**Figure 1:**
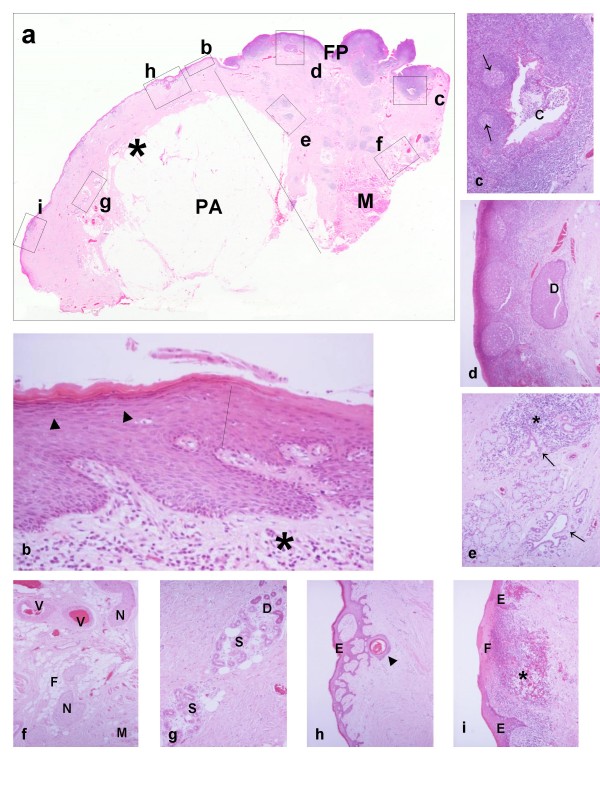
**(a) Scanning view of a skin flap (asterisk) which has been transferred adjacent to lingual foliate papillae (FP)**. The panniculus adiposus (subcutaneous fat, PA), submucosal skeletal muscle (M) and the mammillated surface of the papillae are easily seen. The linear segment indicates the junction between the flap and native mucosa. The areas in rectangles labelled as b-i are magnified in Figs 1b-i respectively, so that particular features are appreciated. **(b) **The linear segment indicates the junction between epidermis (left part of the photomicrograph) and oral epithelium (right part of the photomicrograph). The stratum granulosum (arrowheads) of the epidermis is discernible. The lower border of both epidermis and oral epithelium is irregular. This indicates hyperplasia of rete in response to the presence of non-specific chronic inflammation (asterisk) in underlying stroma. **(c) **Cross-sectioned profile of a crypt (C) of the lingual tonsil, which is surrounded by hyperplastic lymphoid tissue with germinal centres (arrows). Emigrant lymphoid cells and debris are present in the crypt lumen. **(d) **Cross-sectioned profile of a metaplastic collecting duct of the posterior superficial lingual salivary gland (Weber's). Lymphoid tissue with three germinal centres is present at the left of the photomicrograph. It is difficult to distinguish the surface oral epithelium at this magnification. **(e) **Two lobules of the posterior superficial lingual salivary gland, which are variably affected by parenchymal atrophy and non-specific chronic inflammation. Inflammation (asterisk) is prominent in the upper lobule, whereas mucous acini and tubules are preserved in the lower. The arrows indicate ectatic atrophic duct-like structures. **(f) **Randomly mixed fat (F), skeletal muscle (M), nerve fascicles (N) and vessels (V) characterise the submucosal soft tissues and contrast with the uniform and orderly arrangements of panniculus adiposus. **(g) **Two eccrine sweat-gland lobules. Paler secretory segments (S) and darker ducts (D) are distinguished. Adipocytes are mixed with the secretory segments. **(h) **Retiform downward growth of epidermis. The changes resemble senile (solar) lentigo or inchoate seborrhoeic keratosis. The structure indicated by the arrowhead is probably a cross-sectioned plugged infundibulum. Elsewhere, the epidermis of the flap shown in Fig. 1a is attenuated or ulcerated. **(i) **Area of ulceration covered by fibrinous membrane (F). There is underlying non-specific chronic inflammation (asterisk). E, preserved epidermis. Other small ulcerated areas are discernible in Fig. 1a, but these are not shown. Note: Unless otherwise specified, the photomicrographs in this article are from sections of routinely processed tissue, which were stained with haematoxylin and eosin (HE); it was not deemed necessary to give objective magnifications.

**Figure 2:**
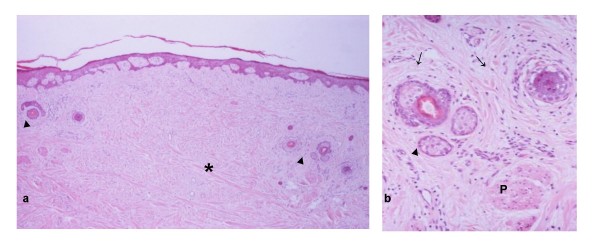
**(a) Part of an intra-orally transferred skin flap includes two "widely separate"' groups of "shrunk" pilosebaceous units**. The randomly arranged collagen bundles (asterisk) of the reticular dermis are seen between the groups. Sweat glands are not present here. **(b) **The pilosebaceous group in the right of Fig. 2a comprises sebaceous glands (arrowhead), arrector pili muscle (P) and hair follicles (arrows) cross-sectioned through their upper (left) or lower (right) segments. Despite the "wide separation" and "shrunk" appearance, the preservation of lower segment suggests that the follicles have not entered the involutionary catagenous stage that would be expected during "mucosalisation".

In cases without active inflammation, cutaneous phenotypes are maintained [7,10]. Epidermal horny and granular layers, eccrine sweat glands and the abundant septate subcutaneous fat are easily identified (Fig. 1). In contrast to the gentle undulating junction between parakeratinised oral mucosa and the lamina propria, the configuration of the epidermal-dermal junction is flattened or shows epithelial rete interdigitating with dermal papillae. Basal keratinocyte pigmentation (freckling) [4] and elastosis may also be present, and occasionally a small melanocytic naevus is included in the flap. Possibly, the features relate to the patient's age and sex and site of the donor flap rather than duration of the skin flap or its site within the mouth. However variation in epidermal thickness is seen, increased thickness being emphasised by some authors [7], and, in contrast to the consistently present sweat glands, pilosebaceous units may appear reduced in number and size (Fig. 2). It is difficult to establish whether such reduction reflects "mucosalisation" or locoregional differences in number and distribution of pilosebaceous units. Nevertheless, blending or gradual transition between the skin flap and the native oral mucosa is usually not seen, the "junctional" zone being abrupt with a sudden loss of the horny and granular layers, a reduction in basal keratinocyte pigmentation, a change in the architecture of the connective tissue [7] and replacement of dermal appendages by minor salivary glands (Fig. 1). In addition, when the skin flap has been transferred to non-masticatory (parakeratinised) areas of the oral mucosa, the junctional zone is easily appreciated in sections stained with periodic acid-Schiff (PAS) because the glycogenated PAS(+) spinous keratinocytes of the parakeratinised oral epithelium sharply contrast with the non-glycogenated keratinocytes of the cutaneous stratum spinosum [4]. Scar tissue may also be present in the junctional zone.

When inflammation is present, the horny layer may be lost and, in most cases, a psoriasiform pattern is seen. This is explained by colonisation of the skin flap surface by *Candida *species. (For illustration, see the following section.) We have observed fibrinous exudate with inflammatory cells and cell debris (pseudomembranous coating) on the epidermis in around 20% of skin-flaps removed during a restructuring procedure, intra-epidermal hyphae in around 40% and involvement of the adjacent oral mucosa in around 50% of skin-infected cases. Hence, candidiasis is a common histological finding even when it is not noted clinically. Acute pseudomembranous and chronic candidiasis is common in oral cancer patients and is attributable to a combination of multiple local and systemic predisposing factors [18]. It is likely that the skin-flap is at greater risk than the native oral mucosa since constant immersion in water is an important risk factor for cutaneous candidiasis [19].

## Chronic hyperplastic candidiasis

Chronic hyperplastic candidiasis (CHC) is the most common histological diagnosis for lesions removed for diagnostic biopsy. Three different reaction patterns occur.

**Figure 3:**
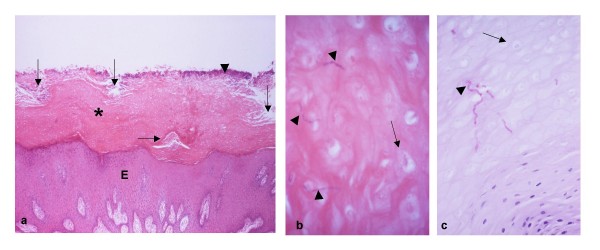
**(a) "Cutaneous" reaction pattern of CHC in a skin flap**. Thick orthokeratotic plaque (asterisk) covers epidermis (E) of increased thickness. Bacterial colonies (arrowhead) are seen on the surface of the plaque. Although the plaque is largely compact, superficial (vertical arrows) and deep (horizontal arrow) areas of variably decreased/lost intercellular cohesion (dyscohesion) are seen. While the superficial dyscohesion leads to desquamation of individual cornified cells, lateral expansion/"burrowing" of deep dyscohesion could eventually effect the loss of the overlying non-dyscohesive cornified heap. The processes would result in formation of surface ditches. **(b) **Even without special staining, candidal hyphae deeply "entombed" within the plaque, are discernible. Note the nuclear "ghosts" of the polygonal cornified keratinocytes. **(c) **The septate and grouped hyphae (arrowheads) are highlighted on PAS-staining. Compare the unstained nuclear ghosts (arrow) with the viable haematoxyphilic nuclei in the right lower corner of the photomicrograph.

Around 50% of cases show a predominantly "cutaneous" reaction pattern with epidermal hyperplasia, acanthosis and marked hyperorthokeratosis (Fig. 3). Intra-epidermal neutrophils and subcorneal spongiform microabscesses are sparse and associated with small foci of parakeratosis while a lympho-plasmacytic infiltrate of variable intensity is seen in the superficial dermis. PAS-staining may reveal grouped candidal hyphae within a desquamating horny layer and sometimes extending into the spinous layer. The direction of the hyphae varies as they penetrate between and within flattened or polygonal keratinocytes and some are lying parallel to the epidermal surface. However, the hyphae may not be immediately apparent and in such cases, surface ditches may be present and should alert the pathologist. The ditches probably result by means of desquamation of large clumps of horny layer and hyphae. Assessment of step-serial sections may be helpful herein.

**Figure 4:**
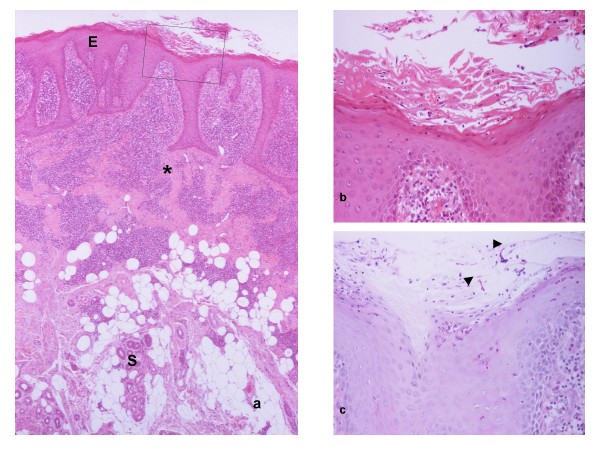
**(a) "Mucosal" reaction pattern of CHC in a skin flap**. The epidermis (E) shows absence of orthokeratinisation, which is difficult to observe at this magnification, variably desquamating parakeratinising layers (area in rectangle) and irregular hyperplasia (acanthosis). Nodular/perivascular chronic inflammation and fibrosis expand the reticular dermis (asterisk). Subcutaneous fat and sweat glands (S) are seen, which allows confident identification of the flap. The area in rectangle is magnified in Fig. 4b. **(b) **The absence of orthokeratinisation and desquamating parakeratinised layers are better appreciated here. **(c) **Adjacent section stained with PAS shows individual hyphae (arrowheads) between desquamating keratinocytes.

**Figure 5:**
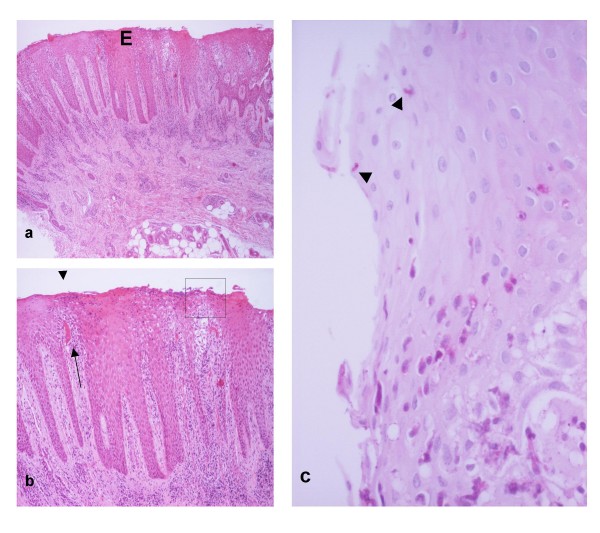
**(a) "Mucosal" reaction pattern of CHC in a skin flap**. Here, the loss of orthokeratinisation and overall appearances of epidermis (E) and papillary dermis could be described as "psoriasiform mucosalisation". Deep dermal and subcutaneous features as in Fig. 4a. **(b) **The "psoriasiform features" are better appreciated on higher magnification. Note the tube-like epidermal rete, attenuated suprapapillary plates (arrowhead) and elongated dermal papillae that contain hyperaemic capillary-sized vessels (arrow). The area in rectangle corresponds to that shown in Fig. 5c. **(c) **This is higher magnification and from an adjacent section stained with PAS. The arrowheads indicate individual hyphae infiltrating parakeratinised layers. The densely stained elements in the lower part of the photomicrograph are emigrating neutrophils.

Around 35% of CHC cases show a predominantly "mucosal" reaction pattern (Figs 4, 5) in which the horny and granular layers are replaced by desquamating layers of parakeratin containing spongiform microabscesses. Here, the rete processes tend to be long with variously bulbous or fused tips while the suprapapillary plates tend to be thin and micro-ulcerated. Candidal hyphae tend to be distributed as individual units rather than grouped and penetrate the superficial parakeratin layers at right angles to the surface.

In the third "mixed" reaction pattern, cutaneous and mucosal patterns are present within different regions of the same lesion in roughly equal proportions. As in the "sub-clinical" infections seen in skin-flaps removed during restructuring procedure and mentioned above, infection of the native oral mucosa is seen in around 50% of CHC cases where the biopsy includes the adjacent oral mucosa.

It seems likely that the range of changes seen in intra-oral skin flaps reflects a similar sequence of events to that occurring in cutaneous candidal infections [20]. While compact hyperorthokeratosis probably characterises lesions of longer duration, the loss of the horny layer and other features of the mucosal reaction pattern seem to represent changes occurring early in the infection. The species of *Candida *involved and differences in the immune response may be further factors in determining the reaction pattern.

The diagnosis of CHC is not always straightforward. The mucosal reaction pattern (Figs 4, 5) is well known to oral pathologists and also, to general pathologists since it is seen in superficial, acute and sub-acute infections of skin. In contrast, the cutaneous reaction pattern with its striking compact hyperorthokeratosis (Fig. 3) can be problematical. In infections of the skin and nail beds, compact hyperorthokeratosis is the hallmark of chronicity [20], but is often unfamiliar to pathologists since chronic cutaneous infections are not common and are often diagnosed by clinical assessment or scrapings rather than biopsy. The grouping of candidal hyphae and their tendency to detach along with clumps of horny layer, and the absence of intra-epithelial microabscesses add to the diagnostic difficulty. Furthermore, the unfamiliarity of compact hyperorthokeratosis in intra-oral biopsy specimens may lead to an erroneous diagnosis. Care is needed also when the rete hyperplasia characterising the mucosal reaction pattern is marked and accompanied by "reactive" cytological atypia since it could be mistaken for microinvasive SCC, but see the following section.

## Chronic candidiasis and squamous epithelial dysplasia

Our experience suggests that around 25% of skin flap lesions diagnosed as CHC show features of squamous epithelial dysplasia, mild or moderate grade. The dysplasia tends to be focal, with sharply defined margins and associated with parakeratosis and marked desquamation. The most conspicuous features are nuclear hyperchromatism, basal cell hyperplasia, drop-shaped rete processes, and in some cases, dyskeratosis affecting all the keratinocytic cell layers. The appearance and degree of the atypia can be such that they cannot readily be explained solely as reactive or regenerative features, and their precise status and prognosis are uncertain. The possibility that the candidal infection is superimposed on an inherent dysplastic lesion must be considered and, hence, re-biopsy after antifungal therapy is advisable. Often, however, the pathogenesis and prognosis remain uncertain and it is important to convey this uncertainty to the clinician. Such lesions have to be managed on an individual basis until the results of long-term follow up studies become available.

There are numerous reports of dysplasia occurring in CHC of the oral mucosa [21-23], but this is not mentioned in accounts of chronic cutaneous candidiasis [20]. The distinction between "reactive" cytological atypia in response to candidal infection and inflammation from inherent dysplasia with malignant potential is difficult. The appearance and degree of the individual cellular and architectural atypia must be considered firstly followed by consideration of the numbers and depth of intra-epithelial hyphae and the intensity of the inflammatory response, and any erosion or ulceration. Nevertheless, the final decision may be subjective and largely based on experience.

## *In-situ *and invasive squamous cell carcinoma

**Figure 6:**
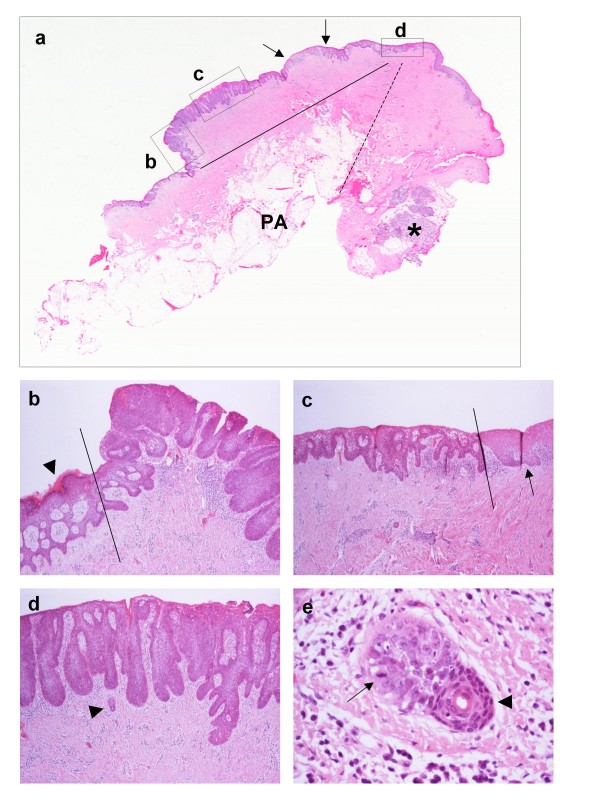
**(a) Scanning view of a "proliferative" carcinoma *in-situ *arising in an intra-orally transferred skin flap**. The continuous linear segment indicates the surface extent of the lesion. The interrupted linear segment indicates the junction between the flap and native mucosa. Note that the carcinoma *in-situ *does not extend to the mucosa. The epidermal area between the arrows is not affected by *in-situ *carcinoma. This is attributable to irregular margins of a single lesion, being not included in the plane of the section, rather than separate lesions. The areas in rectangles labelled as b-d are magnified in Figs 6b-d respectively, so that particular features are appreciated. PA, panniculus adiposus; asterisk, submucosal minor salivary gland. **(b) **The linear segment indicates the junction between carcinoma *in-situ *and epidermis of the flap. While the surface of the carcinoma *in-situ *(right part of the photomicrograph) is gently mammillated and non-keratinised, the epidermis is mildly orthokeratotic (arrowhead) and shows changes similar to those in Fig. 1h. **(c) **The linear segment indicates the junction between carcinoma *in-situ *and native, parakeratinised oral mucosa. The arrow indicates *artefactual *crease of the section. **(d) **The affected epidermis shows absence of keratinisation, full-thickness severe dysplasia and elongated, variously bulbous or fused rete. Although the lower epidermal border is asymmetrical and at different levels, it is smooth and frank invasion is not seen. The increased epidermal thickness accounts for the characterisation of the carcinoma *in-situ *as "proliferative". Dermal inflammation is inconspicuous. The structure indicated by the arrowhead is magnified in Fig. 6e. **(e) **Carcinoma *in-situ *spreads along the lining of an eccrine sweat duct ("eccrinotropism"). The paler, polymorphic and variably dyscohesive malignant keratinocytes, one of which undergoes mitosis (arrow), are easily distinguished from the more compact and densely stained normal ductal lining (arrowhead). The lumen and adluminal cuticle of the duct can be seen.

New primary SCCs of the oral mucosa at the junction of the skin flap are not uncommon [17] and may spread into the peripheral skin flap. In addition, a small proportion of our skin flap biopsies – around 10% – is diagnosed as carcinoma *in-situ *(Fig. 6), papillary or proliferative dysplasia [24] or frank SCC arising from the surface epidermis. That percentage may sound surprising as only sporadic cases are featured in the literature [25]. Possible explanations include our relatively small sample size taken from a population of patients that are largely continuing to smoke and drink; and the clinical expertise of the surgical oncologists, which leads to selective biopsy – hence, 'biased' towards suspicious lesions. The histological appearance of carcinoma *in-situ *is striking and mimics Bowen's disease that typically occurs in sun-exposed areas of fair-skinned older individuals. As in skin, the atypical epithelial cells may involve the pilosebaceous units. In the SCC cases, the tumours are generally well differentiated and keratinising with at least focal areas of acantholytic (adenoid, pseudoglandular) appearance in most and it seems of interest that primary acantholytic SCCs are more common in skin than in oral mucosa [26].

The development of carcinoma *in-situ *and SCC of the skin flap is difficult to explain. It is possible that the skin may have been affected by ultra-violet irradiation or other carcinogens, such as tar, prior to its transfer to the mouth; or the skin flap may have been affected by carcinogens such as tobacco and alcohol following its transfer to the mouth. The role of chronic candidal infection is uncertain. Another possibility is that the epidermis of the skin-flap can become colonised by oral epithelial cells (showing inherent abnormalities) by either a "creeping" or "seeding" mechanism. The creeping mechanism seems unlikely when step sectioning confirms evident and well defined junctional zone between the affected epidermis and oral epithelium, in cases of pathology centrally located within the skin flap and well away from the junctional zone, and when there is no evidence of dysplasia of the native oral mucosa. Creeping, however, may be a factor in lesions occurring at the junctional zone, and in patients with field cancerisation or multiple and multifocal tumours of the native oral mucosa. The seeding mechanism should be considered particularly when erosion/ulceration with florid granulation tissue are noted at the junction of the skin flap and the native oral mucosa (Fig. 1i), there being similarities with  the epithelialisation of dental pulp polyps [27]. The viable desquamated oral keratinocytes showing inherent abnormalities and seeding the granulation tissue could originate from sub-clinical field cancerisation, persistent or residual tumour and a new primary.

## Hyperkeratotic plaque, acanthomas and appendage tumours

A diagnosis of hyperkeratotic plaque is made when there is localised hyperorthokeratosis without evidence of fungal infection, dysplasia or inflammation. The lesion probably represents a frictional callus, due, for example, to denture trauma.

Inchoate or established seborrhoeic keratoses can be seen in around 15% of skin-flap lesions sent for diagnostic biopsy (Fig. 1h), with acanthotic (solid) and hyperkeratotic (papillomatous) patterns in around equal frequency. We have seen one case of adnexal tumour resembling eccrine syringofibroadenoma. It is tempting to speculate that, when the skin flap did not show clinically evident pathology prior its transfer within the mouth, the acanthomas/appendage tumours are secondary to this event. This would suggest that the transferred skin develops and exhibits typical cutaneous histopathology in an "alien" environment, and would be a further argument against mucosalisation. On the other hand, the possibility that subclinical acanthomas/appendage tumours were present in the flap prior to transferring cannot be excluded. This is supported by the aforementioned finding of small melanocytic naevi in the flaps.

## Conclusion

Although skin flaps have been routinely used to reconstruct the oral cavity for more than 20 years, little is known about adaptive events and disease potential. The present review has highlighted areas of uncertainty and drawn attention to the relatively high proportion of biopsies, especially those at the edge of the skin flap, showing squamous epithelial dysplasia or worse. On this basis, it is recommended that clinicians biopsy any lesions where the clinical diagnosisis in doubt and which do not respond fully to antifungal therapy. It is hoped that long-term clinical and histological studies will be undertaken to assess the accuracy of recognising and interpreting infections, reactive processes, and potential and frank malignancies of the transferred epidermis.

## Competing interests

The authors declare that they have no competing interests.

## Authors' contributions

JAW and AT jointly wrote the manuscript, read and approved it. AT prepared the illustrations.

## References

[B1] Soutar DS, McGregor IA (1986). The radial forearm flap in intraoral reconstruction: the experience of 60 consecutive cases. Plast Reconstruct Surg.

[B2] Vaughan ED (1994). The radial forearm flap in orofacial reconstruction. Int J Oral Maxillofac Surg.

[B3] Eliachar I, Sebek BA, Levine S, Tucker HM (1985). Histologic changes in skin implanted into the larynx and trachea by myocutaneous flap reconstruction. Otolaryngol Head Neck Surg.

[B4] Dellon AL, Tarpley TM, Chretien PB (1976). Histologic evaluation of intraoral skin grafts and pedicle flaps in humans. J Oral Surg.

[B5] Beahm EK, Evans GR, Colome-Grimmer MI (1997). Histologic changes of intraoral free skin flaps. Am J Surg.

[B6] Katou F, Motegi K, Tagami H, Shirai N, Echigo S, Nagura H (1999). Unique inflammatory features noted in intraorally transferred skin flaps: correlation with candida albicans infection. Oral Surg Oral Med Oral Pathol Radiol Endod.

[B7] Badran D, Soutar DS, Robertson AG, Reid O, Milne EW, McDonald SW, Scothorne RJ (1998). Behavior of radial forearm skin flaps transplanted into the oral cavity. Clin Anat.

[B8] Shibahara T, Noma H, Takeda E, Hashimoto S (2000). Morphologic changes in forearm flaps of the oral cavity. J Oral Maxillofac Surg.

[B9] Khan AL, Cloke DJ, Hodgkinson PD, McLean NR, Soames JV (2001). Do intraoral radial forearm free flaps re-mucosalise and is candida infection relevant?. Br J Plast Surg.

[B10] Sinclair A, Johnston E, Badran DH, Soutar DS, Robertson AG, McDonald SW (2004). Histological changes in radial forearm skin flaps in the oral cavity. Clin Anat.

[B11] Shibahara T, Mohammed AF, Katkura A, Nomura T (2006). Long-term functional results of free radial forearm flap used for oral reconstruction: functional and histological evaluation. J Oral Maxillofac Surg.

[B12] Rubino C, Dessy LA, Farace F, Ena P, Mazarello V (2002). Microscopic and immunohistochemical analysis of the skin changes of free forearm flaps in intraoral reconstruction. Ann Plast Surg.

[B13] Katou F, Shirai N, Kamakura S, Tagami H, Nagura H, Motegi K (2003). Differential expression of cornified cell precursors in normal skin, intraorally transplanted skin and normal oral mucosa. Br J Dermatol.

[B14] Reichart P, Lonning T, Hausamen JE, Becker J (1984). Morphologic study of jejunal mucosal transplants for the replacement of oral mucosa. J Oral Pathol.

[B15] Krikos GA (1971). Dermo-epidermal interactions. Oral Surg Oral Med Oral Pathol.

[B16] Wei WI, Lam KH, Lau WF (1989). Fate of skin element of pectoralis major flap in intraoral reconstruction. Head Neck.

[B17] Woolgar JA, Rogers S, West CR, Errington RD, Brown JS, Vaughan ED (1999). Survival and patterns of recurrence in 200 oral cancer patients treated by radical surgery and neck dissection. Oral Oncology.

[B18] Reichart PA, Samaranayake LP, Philipsen HP (2000). Pathology and clinical correlates in oral candidiasis and its variants: a review. Oral Diseases.

[B19] Odds FC (1994). Pathogenesis of *Candidal *infections. J Am Acad Dermatol.

[B20] Ollague J, Ackerman AB (1982). Compact hyperorthokeratosis as a clue to chronic dermatophytosis and candidiasis. Am J Dermatopathol.

[B21] Cawson RA, Binnie WH, Mackenzie IC, Dabelsteen E, Squier CA (1980). Candida leukoplakia and carcinoma: a possible relationship. Oral premalignancy Proceedings of the first Dow Symposium Iowa.

[B22] Reibel J (2003). Prognosis of oral pre-malignant lesions: significance of clinical, histopathological, and molecular biological characteristics. Crit Rev Oral Biol Med.

[B23] McCullough M, Jaber M, Barrett AW, Bain L, Speight PM, Porter SR (2002). Oral yeast carriage correlates with presence of oral epithelial dysplasia. Oral Oncol.

[B24] Woolgar JA, Triantafyllou A (2008). Pitfalls and procedures in the histopathological diagnosis of oral and oropharyngeal squamous cell carcinoma and a review of the role of pathology in prognosis. Oral Oncol.

[B25] Sa'do B, Nakamura N, Higuchi Y, Ozeki S, Harada H, Tashiro H (1994). Squamous cell carcinoma of the oral cavity derived from a skin graft: a case report. Head & Neck.

[B26] Cassarino DS, Derienzo DP, Barr RJ (2006). Cutaneous squamous cell carcinoma: a comprehensive clinico-pathologic classification. Part one. J Cutan Pathol.

[B27] Southam JC, Hodson JJ (1974). The growth of epithelium, melanocytes, and Langerhans cells on human and experimental dental pulp polyps. Oral Surg Oral Med Oral Pathol.

